# Graphene Oxide as a Multifunctional Platform for Intracellular Delivery, Imaging, and Cancer Sensing

**DOI:** 10.1038/s41598-018-36617-4

**Published:** 2019-01-23

**Authors:** E. Campbell, Md. Tanvir Hasan, Christine Pho, K. Callaghan, G. R. Akkaraju, A. V. Naumov

**Affiliations:** 1 Department of Physics and Astronomy, Texas Christian University, Fort Worth, TX 76129 USA; 2 Department of Biology, Texas Christian University, Fort Worth, TX 76129 USA

## Abstract

Graphene oxide (GO), the most common derivative of graphene, is an exceptional nanomaterial that possesses multiple physical properties critical for biomedical applications. GO exhibits pH-dependent fluorescence emission in the visible/near-infrared, providing a possibility of molecular imaging and pH-sensing. It is also water soluble and has a substantial platform for functionalization, allowing for the delivery of multiple therapeutics. GO physical properties are modified to enhance cellular internalization, producing fluorescent nanoflakes with low (<15%) cytotoxicity at the imaging concentrations of 15 μg/mL. As a result, at lower flake sizes GO rapidly internalizes into HeLa cells with the following 70% fluorescence based clearance at 24 h, assessed by its characteristic emission in red/near-IR. pH-dependence of GO emission is utilized to provide the sensing of acidic extracellular environments of cancer cells. The results demonstrate diminishing green/red (550/630 nm) fluorescence intensity ratios for HeLa and MCF-7 cancer cells in comparison to HEK-293 healthy cells suggesting a potential use of GO as a non-invasive optical sensor for cancer microenvironments. The results of this work demonstrate the potential of GO as a novel multifunctional platform for therapeutic delivery, biological imaging and cancer sensing.

## Introduction

Graphene is utilized in a number of applications, such as water desalination^1^, new age electronics^2,3^, graphene-assisted laser desorption/ionization for mass spectrometry^4^ and high resolution electron microscopy^5^, due to its unique electrical^6^, thermal^7^ conductivity, tensile strength^8^ and transparency properties^9^. Recently graphene has been used in biomedical applications including DNA sequencing^10^, biosensor development^11^, and graphene-enhanced cell differentiation and growth^11^. As graphene is insoluble in water, such applications are limited to passive platforms for sensing and cell work. Its functional derivative graphene oxide (GO) possesses unique properties which make it more attractive for biomedical application: it is water soluble, provides a large platform with a variety of addends for convenient functionalization-based drug attachment, and exhibits fluorescence in visible/near-infrared spectrum. These properties are utilized in GO field-effect transistor, biosensors^12–14^, cellular probing and real-time monitoring via GO nanosheets using a wide-field fluorescence microscope^15^, and scaffolding for cell cultures and tissue engineering^16^. Nanoscale graphene oxide has also been adopted for the delivery of anticancer drugs into biological cells^12^^,17–19^, aptamers for ATP probing in mouse epithelial cells, and gene delivery^15,20,21^. For such applications, however, GO was modified and has only been utilized as a delivery agent or rarely as a fluorescence marker^16,18^ requiring either incorporation of external fluorophores^22–24^ or complementary covalent functionalization with PEG for successful delivery^24,25^. Additionally, many GO forms used in biological applications exhibit intrinsic fluorescence in the visible^26,27^ even with advantageous near-IR 2-photon excitation^28^. This can be optimal for *in-vitro* work or low penetration depth imaging but not for conventional *in-vivo* studies where near-IR emission in the water window is desired for deep tissue penetration. All these complexities hamper the potential use of GO in biomedical applications resulting in the lack of data on its cytotoxicity and non-targeted intracellular accumulation. Finally, *in-vitro* optical sensing capacity of GO has not been utilized to date. This study fills the aforementioned gap by exploring the properties of GO as a standalone multifunctional agent for imaging in red/near-IR, cellular internalization, and biosensing.

The adaptability and variability of medical conditions such as cancer requires both detection and treatment which can only be accomplished by a multimodal approach. The potential to perform multiple functions using one agent is the attractive force driving the integration of molecular cancer therapeutics with nanomaterials-based drug-delivery vehicle systems^29^. Many such nanoformulations are currently used for drug transport and imaging^30–32^, however few possess concomitant sensing capacity^33,34^. Cancer detection is critical for effective therapeutics as it would allow for early treatment, development of the most effective therapeutic plans for patients, as well as for the opportunity to advance cancer research^35–37^. Thus, development of molecular cancer sensors is of high priority in the field of cancer therapeutics. Cancer cells excrete lactic acid^38^ as a result of which cancerous environments have low pH values^39,40^. In this work we utilize GO fluorescing in visible/NIR and explore the property of GO to vary its fluorescence as a function of pH^41^ in the biological range for the detection of such cancerous environments. Additionally, to detection, GO can facilitate improved treatment: protecting gene therapeutics from nuclease-mediated degradation^42–44^ or enhancing local therapeutic concentrations via intracellular delivery of the drugs that can be attached to it via a variety of functionalization approaches covalently and non-covalently^45^.

Graphene oxide is proposed as a prospective platform for therapeutics, as unlike other therapeutic nanoformulations, it can be produced at low cost and large quantities, easily functionalized with therapeutics, and exhibits pH-dependent fluorescence in the red/near-IR spectral region with reduced biological autofluorescence background and tissue scattering. The feasibility of *in-vitro* GO optical pH sensing is investigated as a novel multifunctional agent for delivery, imaging and sensing of cancerous environments. The objective of this work is to optimize GO for these applications and test its feasibility *in vitro*.

## Results

### *In-vitro* imaging

Individual GO flakes can be readily seen in aqueous suspensions (Fig. S1). 480 nm excitation is used throughout this work, while detecting GO emission in red (630 nm) and green (550 nm) to achieve imaging and pH sensing of cancer cell environments. Excitation and emission wavelength ranges are selected on the basis of the spectral analysis of GO emission features and its excitation spectra^46^. Within those ranges, a most optimal combination of excitation and emission filters (535 nm for excitation and 630 nm for emission) yielding the highest intensity of GO emission with lowered autofluorescence from GO-transfected HeLa cells in red was chosen experimentally (Fig. S2). Furthermore, the integration time is reduced to diminish the remaining autofluorescence below the background level.

**Figure 1:**
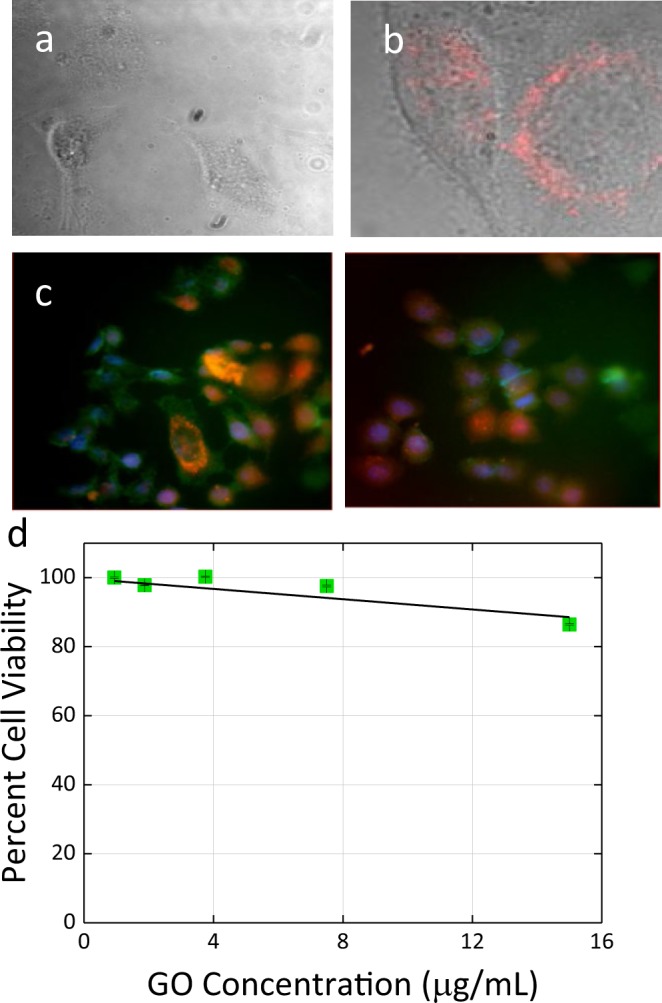
Fluorescence microscopy. (**a**) Non-treatment control HeLa cells. (**b**) HeLa cells transfected with GO. (**c**) Two fluorescence colocalization images of GO emission (red) with DAPI (blue) and Lysotracker Green (green) staining within HeLa cells taken in different regions of the same sample. (**d**) Cytotoxicity of GO in HeLa cells showing percent cell viability with respect to the GO concentration (error bars are below the size of the points on the graph).

As GO was introduced to HeLa cells its ability to internalize was further verified via fluorescence microscopy at 1 h post transfection. Microscopy images of GO-treated HeLa cells washed prior to imaging to remove any extracellular GO adhering to the cell membrane, indicate substantial 630 nm emission from nanoscale GO flakes inside the cells with no apparent autofluorescence in control HeLa cells (Fig. 1a,b). Individual GO flake structures are not resolved, as internalized flake dimensions are expected to be under 300 nm^47,48^. At the same imaging conditions, non-treated cells show no observable emission. DAPI and lysotracker green co-staining shows in Fig. 1c that GO emission does partially co-localize with cell nuclei; additionally, in a number of cells it appears to co-localize with lysosomes stained by lysotracker green, suggesting endocytosis as one of the pathways of cellular entry (Figs S4 and S5) and initial internalization of GO flakes within the lysosomes. GO also shows only small to negligible cytotoxic response at the imaging concentrations as indicated by the MTT assay (Fig. 1d), making it superior to other carbon nanomaterials which are hampered by their profound cytotoxicity^49^.

**Figure 2:**
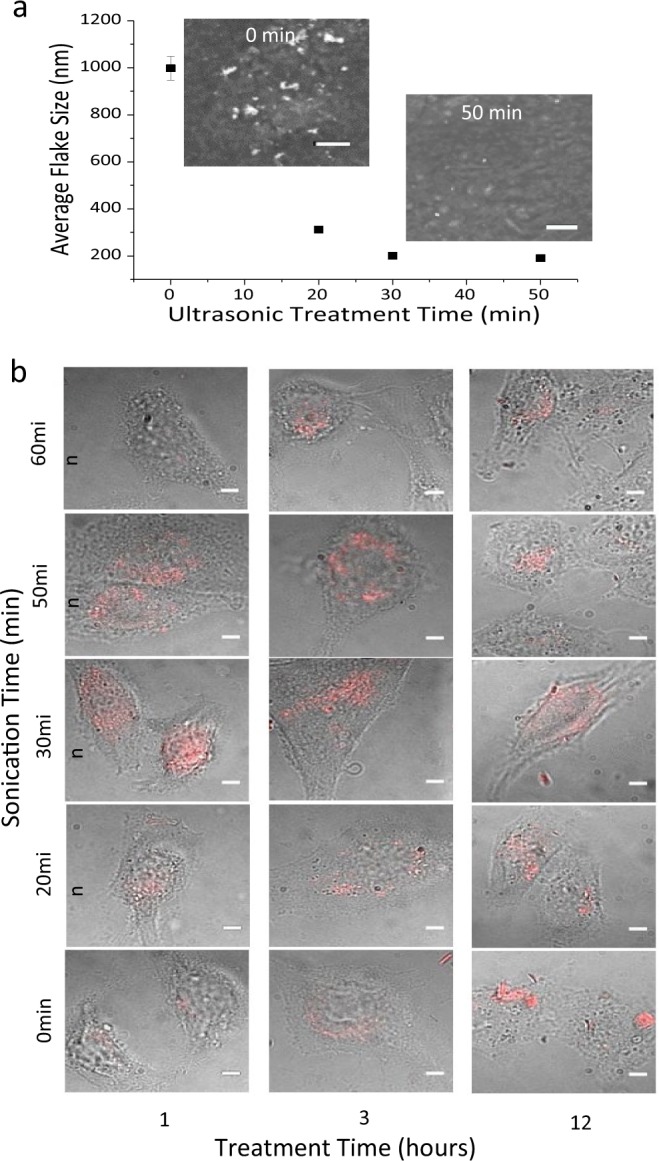
(**a**) Mean Flake Size vs. ultrasonic treatment time from SEM analysis. Insets are SEM images of GO flakes subject to 0 min and 50 min ultrasonic treatment. (**b**) Image matrix of GO emission in HeLa cells with varying ultrasonic treatment time (vertical) vs. transfection time (horizontal). Scale bar length is 5 μm.

Another advantage of GO as a delivery vehicle is its ease of modification, starting from the amount^50,51^ and type^46^ of functional groups, to the size of GO flakes^52^. To achieve optimal internalization and imaging conditions, the influence of transfection time and the size of GO flakes on the internalization efficiency is explored. The mean size of GO flakes is varied by high power ultrasonic processing for periods up to 50 minutes from approximately 1 μm (flake dimensions are measured along their longest axis) down to 190 nm (Fig. 2a) with the expectation that small flakes, sized below 200 nm, will show improved cell penetration^53,54^. This processing does not significantly affect GO fluorescence emission retaining its properties as an imaging agent (Fig. S6). Single-layer flake thickness identified in the atomic force microscopy (AFM) images provided by commercial GO supplier was verified by scanning electron microscopy (SEM) imaging (Fig. S3) to ensure capabilities for successful internalization.

### Cell Internalization

Emission from GO formulations processed by different ultrasonic treatment routines is observed in HeLa cells at 1, 3 and 12 hours post transfection (Fig. 2b).

GO externally attached to cell membrane is removed by two consecutive washing procedures leaving mainly the emission from internalized flakes. The matrix of images for different ultrasonic treatments versus transfection times shows the highest processed by 30 and 50 min of ultrasonic treatment (corresponding flake sizes of 202 nm and 190 nm).

**Figure 3:**
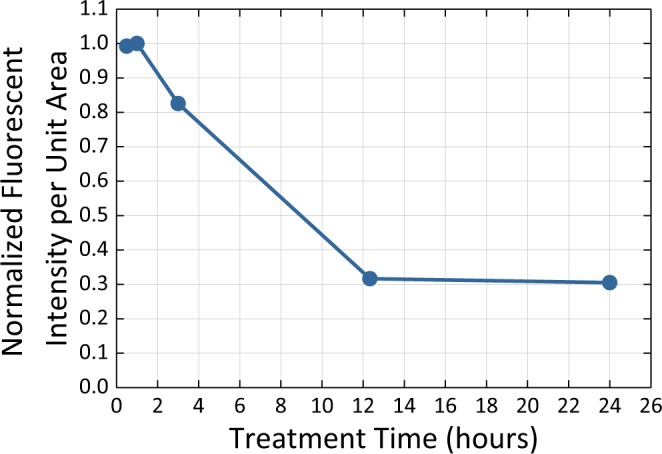
Intensity per unit area of GO emission from HeLa cells depending on the treatment time. Error bars are within the size of the points.

The assessment of the intensity of GO intracellular emission allows comparing the relative concentration of GO flakes in cells at different time points post transfection. As indicated by the plot of integrated fluorescence intensity per cell (Fig. 3), for 30 min-processed GO the optimal internalization occurs at 1 hr post transfection with the following excretion of GO from the cells down to 30% in 24 hrs.

### pH-sensing

**Figure 4:**
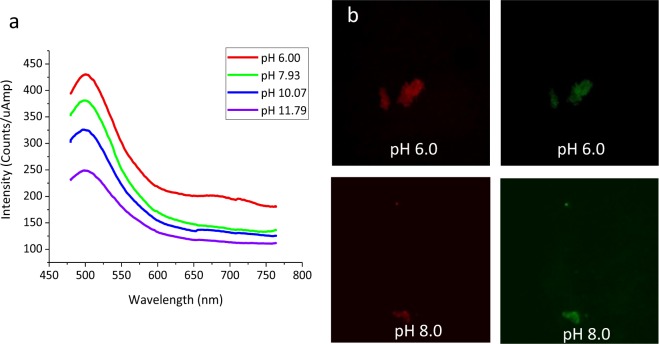
(**a**) Fluorescence spectra of GO in aqueous suspension at varying pH levels. (**b**) Fluorescence of individual GO flakes at pH 6 vs 8 in red (630 nm) versus green (550 nm) with 480 nm excitation.

Since we envision GO as a multimodal agent for imaging, delivery, and sensing, we further explore its capabilities as a molecular pH sensor for cancer detection. This is allowed by pH-dependence of GO emission in the biological pH range with significant changes between pH 6 and 8 (Fig. 4a). Since cancer cells excrete lactic acid, tumor regions maintain acidic environments at approximately pH 6 at which GO shows a markedly different emission signature than at a regular pH of 7–8^38–40^. As pH changes from 6 to 8 (Fig. 4a) the ratio of GO fluorescence in red versus green decreases by the factor of approximately 1.4. This makes GO a potential sensor for acidic cancerous environments. At the single flake level (Fig. 4b), a brighter emission in red (630 nm) than green (550 nm) can be seen for the more acidic (pH 6) environments, and a quenched red emission for more basic (pH 8) environments.

Quantitatively this results in average green to red emission intensity ratios of 0.548 and 0.777 calculated per unit area for pH 6 and 8, respectively for over 400 individual flakes. The difference in emission on the single flake level leads us to assume that such sensing will be possible within cellular environments. To test this hypothesis, GO suspension subjected to 30-minute ultrasonic processing (optimal for internalization) is introduced to two cancer and one healthy cell line (HeLa, MCF7, and HEK-293) without washing to retain extracellular GO.

Statistical analysis of scattered GO flakes located in the intracellular and extracellular environments can be found in Table 1. Due to internal cell buffering, there is little difference for intracellular environments, however GO exhibits substantial extracellular differences in green/red emission ratios. Analysis of GO flakes in the extracellular environment show that green/red ratios of intensity per unit area are greater for healthy rather than cancer cells (Table 1) even though a degree of pH buffering by the media could hamper that determination. Nevertheless, 13–25% relative difference between cancer and healthy cells, provides a significant variation to be used for the detection of cancerous environments, given only 2% difference between such ratios intracellularly and the large sampling size of over 500 flakes.

**Table 1:** Comparison of intracellular vs. extracellular green/red intensity ratios across healthy (HEK-293) versus cancer (HeLa and MCF-7) cell lines, and images demonstrating fluorescence differences for extracellular environments: emission in red is brighter for cancer cells. Scale bar = 5 μm.

550/630 nm Intensity Ratios			
	HEK-293	HELA	MCF-7
INTRACELLULAR	0.929	0.908	0.905
EXTRACELLULAR	0.822	0.622	0.717
Green			
Red			

## Discussion

As a fluorophore, graphene oxide exhibits fluorescence with a quantum yield of approximately 1%^46^, though some report quantum yields of up to 10% in the visible^55^. Few GO materials also exhibit emission near-IR where the biological autofluorescence background is diminished. This makes red/near-IR emissive GO used in the present study an advantageous agent for biological imaging. In this work, GO is optimized and tested for that application. GO-based imaging in HeLa cells (Fig. 1) provides a unique capability not only to track GO and potentially its payload but additionally to assess the internalization and excretion of GO from the cells. Considering that no photobleaching was recorded for individual GO flakes (Fig. S1) over time, increase of GO emission in cells is attributed to internalization, while decrease - to excretion. As more effective internalization with smaller nanoparticle size is expected, the alteration of GO size by ultrasonic treatment allows for more efficient internalization. As evident from the internalization studies in Fig. 2, GO rapidly enters the cells with optimal flake size of approximately 200 nm achieved by 30 to 50 minutes of ultrasonic treatment at 3 W power. This proof of cellular internalization suggests GO as an imaging and potentially a drug delivery agent.

The most extensive ultrasonic treatment (60 min), yields substantial aggregation of GO flakes seen in SEM images (Fig. S7–60 minutes), which hampers successful internalization leading to low intracellular GO emission (Fig. 2b) thus setting a limit of 190 nm for effective flake sizes used in this work. The general trend in this study shows less emission at 12 hours for smaller flake sizes, which indicates potential excretion of graphene oxide from the cells. The optimal flake size (average long axis dimension of 202 nm corresponding to 30 min ultrasonic treatment time), is utilized to determine the optimal internalization/excretion time frame for GO as a therapeutic carrier over time periods of 30 min to 24 hr. The integrated emission intensity per unit cell area maximum for 1 hr post transfection indicates that the highest GO internalization occurs within this minimal time frame further followed by excretion. Significantly slower clearance at later time points could be attributed to size-dependent excretion with smaller flakes excreting at earlier time points while larger flakes take more time to excrete. However, based on the MTT assay (Fig. 1d) the remaining 30% of the initial GO concentration is expected to decrease cell viability only by several percent, suggesting low toxic effect of the residual GO. Although describing the clearance of GO nanoflakes from the cells, this study does not yet provide the information on the clearance from the tissues^56^.

The optimized GO flakes show the capacity of pH-based detection of cancerous environments. Since GO emission spectra (Fig. 4a) depend significantly on pH of the medium, GO has a potential to serve as a non-destructive sensor of the cancerous environments. As the pH of the intracellular environment is not expected to vary greatly due to the internal cell buffering capacity^57,58^, very little differences in green/red ratios of intensity per unit area are seen for intracellular GO emission in either cell type (Table 1). This effect is dictated by pH buffering of cell proteins and phosphate buffers as they restrict the pH in intracellular compartments to a narrow range of approximately 7.1–7.2^58,59^. However, extracellularly green/red GO emission ratios exhibit substantial differentiation between the healthy and cancer cell environment with 20–30% difference between cancer and healthy cells. This suggests a promising potential of GO as a nanoscale local sensor of cancerous environments either *in-vitro*, *ex-vivo* or intravitally using techniques developed for protein sensing^60^ or for determination of tumor borders in surgical procedures^61^. The pH-sensing capacity of GO can be also utilized for applications other than cancer such as detection of microscopic pH changes in media, drug screenings, and assessment of cellular processes^62^^,63^.

Even though further oxidation with ozone treatment could be used to alter the electronic energy transitions of GO and affect the fluorescence peak position, width, and shape^64^, unaltered GO is used in this work, because oxidized GO spectra are shifted to green (Fig. S8) providing less deterministic sensing with fewer visible changes due to pH (acidic and basic features overlap in green). As a result, the unique properties of GO flakes optimized in this work lead to the capabilities of cellular internalization and excretion, fluorescence imaging and cancer detection combined in one molecular platform. Such multifunctional systems provide a novel avenue for cancer therapeutic drug/gene delivery, cancer treatment and diagnostics.

In conclusion, graphene oxide can be used as a multifunctional imaging, delivery, and cancer-sensing agent. GO used in this work is non-toxic at the imaging doses, which opens a possibility for further animal studies with this material. The most efficient cellular internalization of GO occurs at 1 hour post transfection and at the smaller flake sizes of approximately 200 nm that can be simply achieved by 30-minute ultrasonic treatment. In this form, GO can be used as a delivery agent that internalizes quickly and is excreted thereafter with 30% of the maximum cellular intake left after 24 hours. pH-sensitive GO emission not only allows to detect its presence in biological cells, but also provides the means to assess the microscopic pH of the cellular environments. GO showed efficient discrimination of acidic extracellular cancerous environments of HeLa and MCF-7 cells as opposed to healthy HEK-293 cells with weaker differentiation between cell types intracellularly.

This outlines a promising potential of GO as a new candidate for cancer treatment via delivered drug or gene therapeutics, biological imaging via its intrinsic fluorescence in red/near-IR and detection of cancerous environments *in-vitro/ex-vivo* and intravitally. GO offers a fully multifunctional affordable in mass production alternative to existing nanocarriers without the need of attaching additional imaging and sensing moieties that contribute to the toxic profile of the formulation. Additionally, its modifiable platform allows for further variation of GO flake sizes, functional group types and degrees of oxidation allowing to tailor this multifunctional imaging/sensing platform to a variety of applications including detection of enzymatic reactions, glucose or DNA detection and microscopic optical pH sensing for multi-analyte monitoring and sensor arrays.

## Methods

### Sample Preparation

Stock aqueous graphene oxide suspensions were prepared through the dilution of 1.67 mL of commercially available aqueous GO (GoGraphene) to 15 μg/mL. This solution was then subjected to 20 minutes of high power ultrasonic treatment to disperse GO and break apart aggregated flakes. After ultrasonic treatment, the samples were centrifuged for 5 minutes at 2000 × G to remove any remaining aggregates. Further timed high power tip ultrasonic treatment for 20 to 60 min coupled with centrifugation-assisted separation of larger GO flakes allowed for the alteration of average GO flake dimensions in suspension. At that point, GO concentration was assessed via optical absorption measurements based on the standard of known concentration. Select GO samples were subject to mild ozone oxidation^46^ to adjust spectral signatures and enhance emission intensities.

### Optical measurements and characterization of GO samples

Horiba Scientific, SPEX NanoLog fluorescence spectrophotometer was used to generate GO emission spectra with excitation of 400 nm utilized in previous works^46^. Absorbance spectra were measured with Agilent Technologies, Cary 60 UV–vis spectrometer in the range of 200–800 nm to assess sample concentration, with an extinction coefficient of 44.26 mL/μg*cm determined experimentally. NT-MDT Nano Solver AFM and JEOL, JSM-7100F SEM were utilized to measure the average flake size of GO samples ultrasonically processed for 0, 20, 30, 50 and 60 min respectively.

### Cell Culture

Two cancer cell lines (HeLa –Human cervical carcinoma, and MCF-7 – Human breast cancer) were used in this work, as well as one non-cancer cell line (HEK-293, Human embryonic kidney fibroblast). Cells were maintained in a Thermo-Scientific Midi 40 CO_2_ Incubator at 37.1 °C with 5% carbon dioxide, 95% air. For microscopy, glass coverslips were placed at the bottom of 6-well plates, and cells were added afterwards. A minimum of 4 hours was allowed for cell attachment to the coverslips before the addition of GO. GO was introduced to the wells at a final concentration of 15 μg/mL in each well. For the initial test to determine the desired flake size, treatment times of 1, 3, and 12 hours were used. The cells for this experiment were washed with 0.5 mL of PBS and then fixed with 4% paraformaldehyde, RT, 30 min, to remove extracellular GO and prepare cells for imaging. After 30 minutes, the cell sample was washed again with 0.5 mL of PBS and then imaged. For the cell internalization/excretion experiment, 0.5, 1, 3, 12.33, and 24 hours were used as transfection time points. Lastly, for the pH study, cells were imaged without a washing step, to maintain GO presence in both the intracellular and extracellular environments.

### Imaging

Samples were sealed and imaged via visible/near-IR spectrally resolved microscopy setup. A Hamamatsu ImagEM EMCCD camera within the Olympus IX73 microscope setup with 60x IR-corrected Olympus Plan Apo objective was used to image individual GO flakes in aqueous suspensions and *in-vitro*. An optimal configuration of excitation (480 ± 25 nm) and emission (630 ± 12.5 nm as well as 550 ± 20 nm) filters was assessed for GO used in our work and utilized in these experiments. Appropriate exposure time and illumination levels were determined using control cells with no GO present, ensuring zero autofluorescence background.

Fluorescence staining of HeLa cells was performed with DAPI and Lysotracker Green for col-localization study of GO emission. For imaging the following excitation/emission wavelengths were used: GO excitation: 540 nm, emission: 650 nm; DAPI excitation: 375 nm, emission: 450 nm; Lysotracker green excitation: 475 nm, emission: 535 nm.

### Image Analysis

ImageJ software was used for image analysis including calculations of background-subtracted emission per unit area and per biological cell. Background intensity per unit area was calculated by taking an average of the mean gray value of the background and multiplying it by the area of each measured region. Corrected total cell fluorescence (CTCF) was determined by taking the integrated intensity over the whole cell and subtracting out the average background intensity determined from three different background intensity measurements for each image. The statistics of GO emission in green (550 nm) and red (630 nm) for the pH analysis of cancer versus healthy cells was obtained by performing measurements on over 100 cells for each excitation wavelength. The images taken were of the same cells, allowing for the analysis of the exact same objects in both red and green. Again, CTCF was determined by highlighting the fluorescent regions in red and green and subtracting out the average background intensity in the area equal to that of the highlighted regions. The CTCF for red and green GO emission were then compared, allowing for the quantification of the green to red emission ratios.

## Electronic supplementary material

Supplementary Information

Graphene Oxide as a Multifunctional Platform for Intracellular Delivery, Imaging, and Cancer Sensing
