# Supplementary material for: Graphene Oxide as a Multifunctional Platform for Intracellular Delivery, Imaging, and Cancer Sensing
Source: Sci Rep. 2019 Jan 23;9:416. doi: 10.1038/s41598-018-36617-4 (PMC6344482; doi:10.1038/s41598-018-36617-4)
Supplement: Supplementary file 1 — Supplementary Information [file 41598_2018_36617_MOESM1_ESM.docx]

**Graphene Oxide as a Multifunctional Platform for Intracellular Delivery, Imaging, and Cancer Sensing**

E. Campbell,^1^ Md. Tanvir Hasan, ^1^ Christine Pho,^1^ K. Callaghan,^2^ G.R. Akkaraju,^2^ and A. V. Naumov^1^

*1. Department of Physics and Astronomy, Texas Christian University, Fort Worth, TX 76129 USA*

*2. Department of Biology, Texas Christian University, Fort Worth, TX 76129, USA*

**Supporting Information**

***S1*** *video of emission from GO flakes floating in solution under the microscope.*


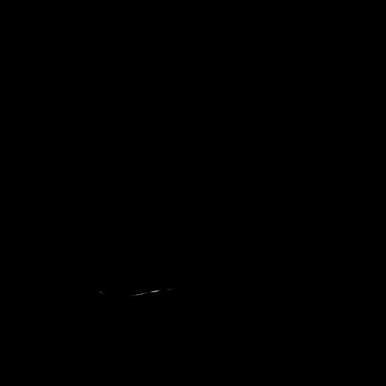


A


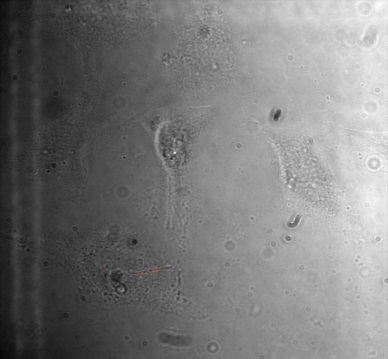


B


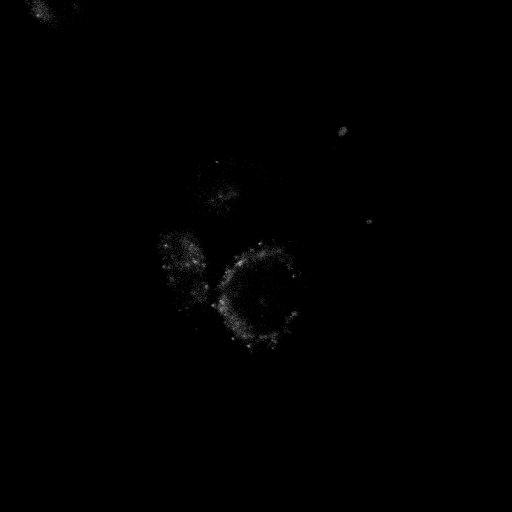


C


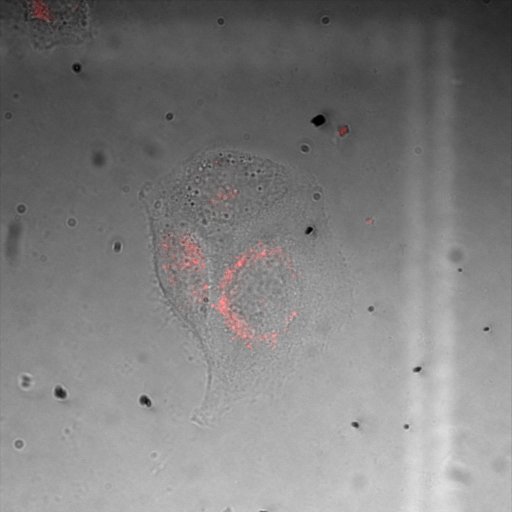


D

***S2*** *A: Fluorescence image of control in HeLa cells B: Bright-field and fluorescence overlay image of control C: Fluorescence image of GO sample within HeLa cells. D: Bright-field and fluorescence overlay images of GO sample within cells.*


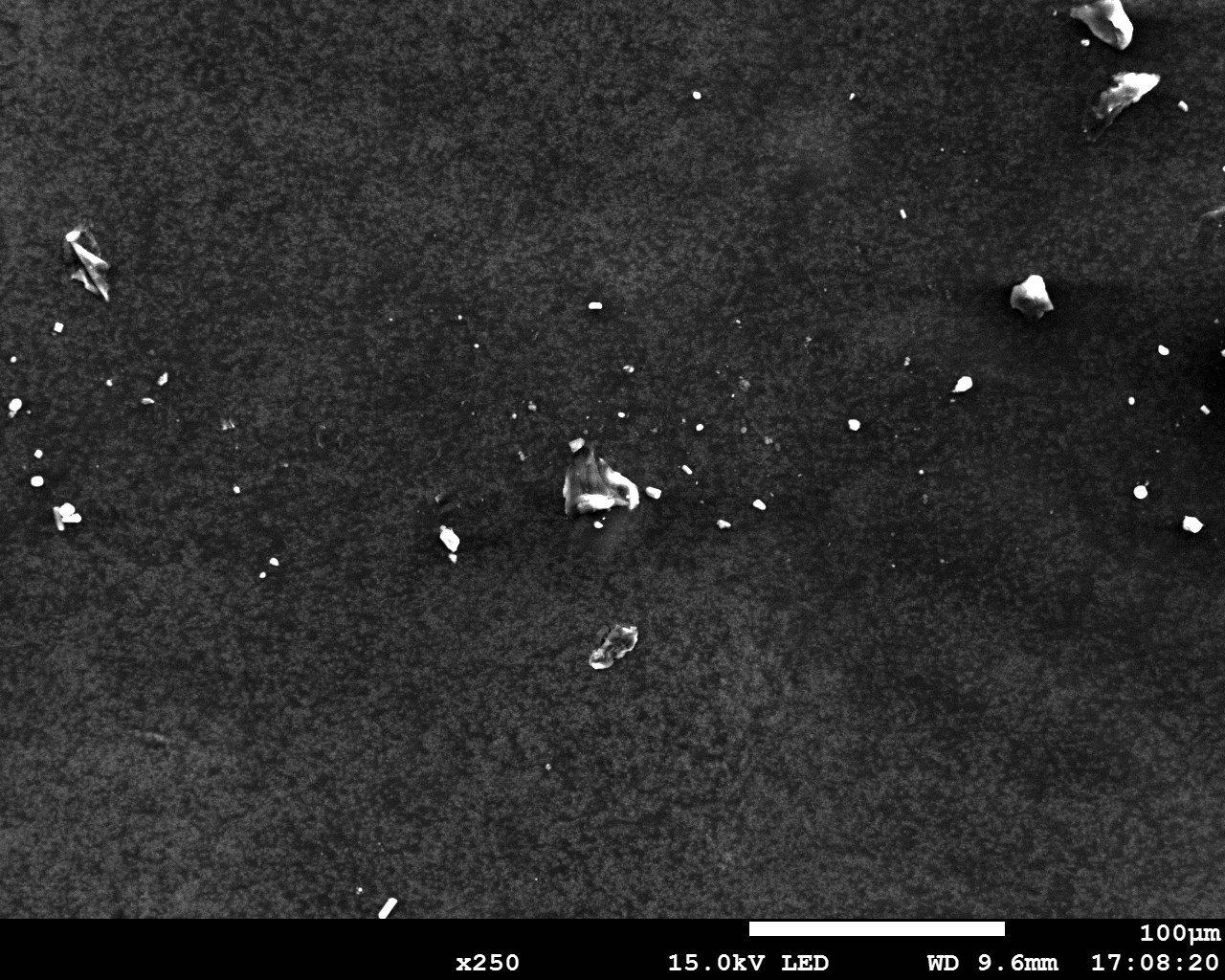


***S3*** *SEM image of single layer GO*


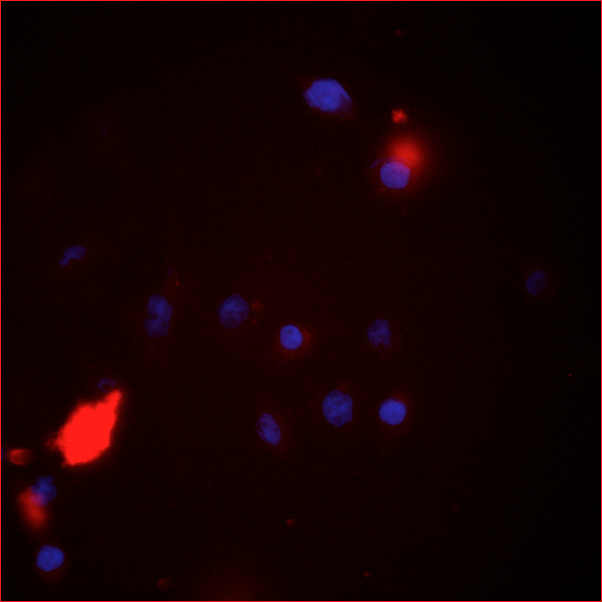

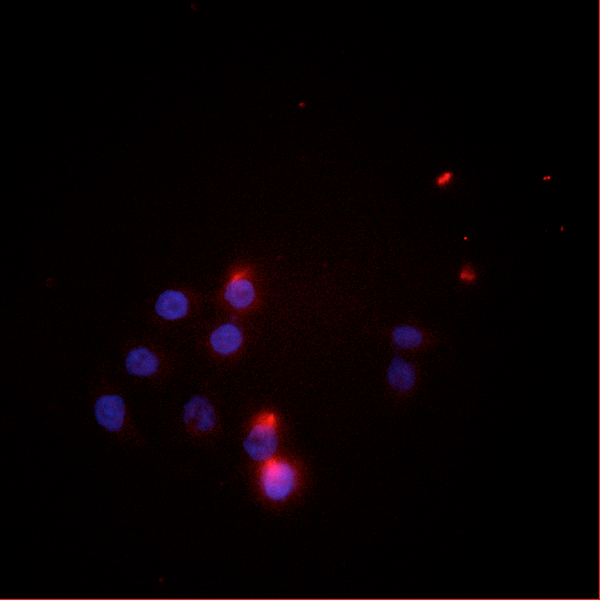


***S4*** *Fluorescence imaging: GO + DAPI staining of HeLa cells*

*
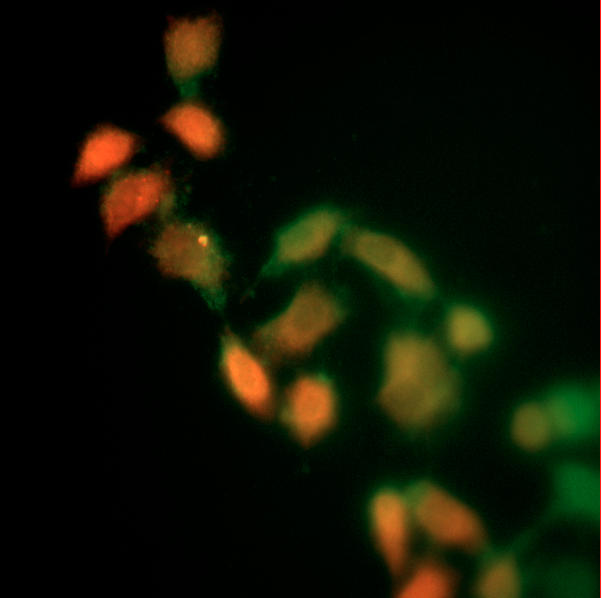

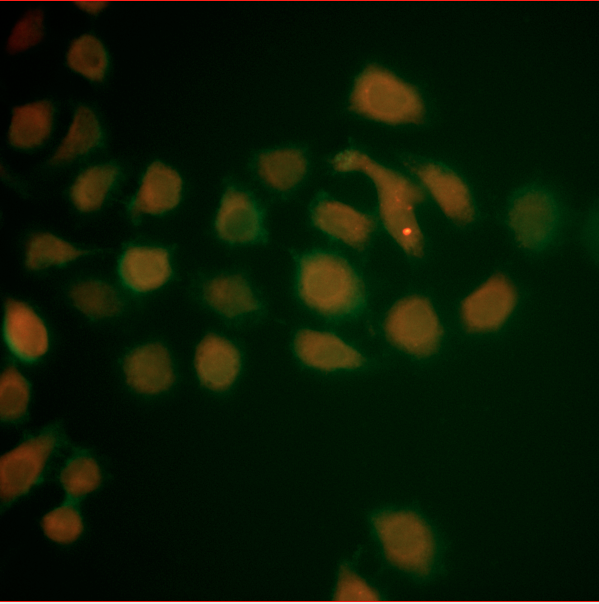
*

***S5*** *Fluorescence imaging: GO + Lysotracker green staining of HeLa cells*


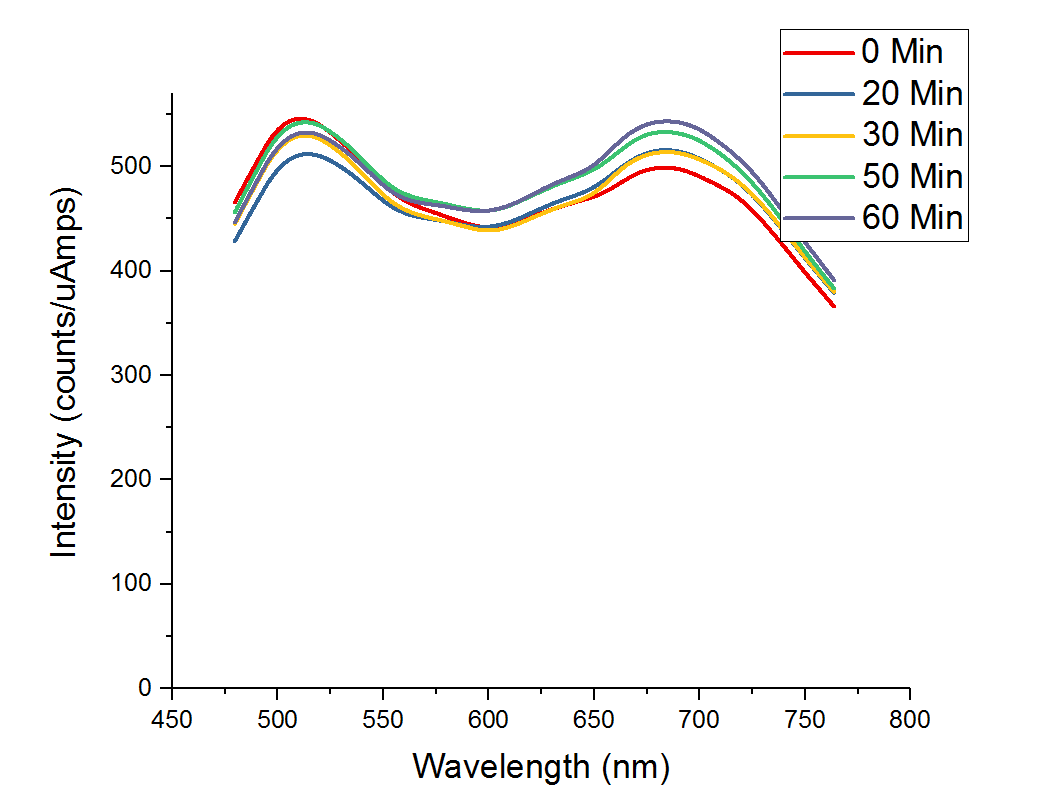


***S6*** *Fluorescence spectra of varying ultra-sonication treatment procedures.*


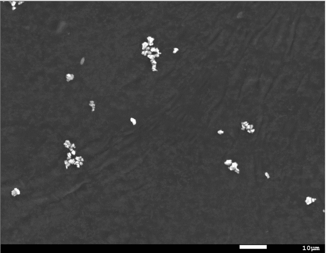

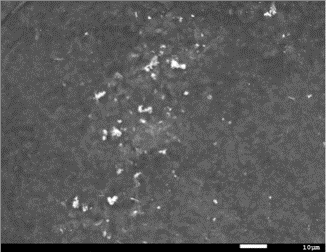

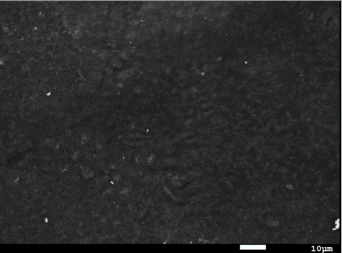

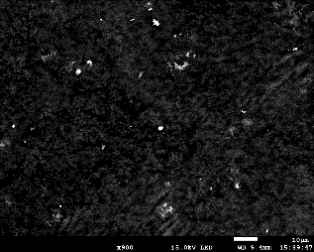


0 min.

20 min.

50 min.

60 min.

***S7*** *SEM images of GO flakes for varying time of ultrasonic treatment. Scale bar is 10 μm.*

***S8*** *Emission spectra of ozone-treated GO.*
